# Early Evaluation of the Short Physical Performance Battery in Hospitalized Patients with Chronic Kidney Disease Predicts Long-Term Hospitalization

**DOI:** 10.3390/diseases13030088

**Published:** 2025-03-19

**Authors:** Takashi Amari, Eiji Kubo, Yota Kuramochi, Shota Onoda, Kyosuke Fukuda, Emi Yokoyama, Masami Kimura, Tomoyuki Arai

**Affiliations:** 1 Department of Physical Therapy, Saitama Medical University, Kaswakado, Moroyamacho, Saitama 350-0496, Japan; arai_tm@saitama-med.ac.jp; 2 Department of Nephrology, Sakura Memorial Hospital, Mizutani Higashi, Fujimi-Shi, Saitama 354-0013, Japan; ek81zzz@gmail.com; 3 Department of Rehabilitation, Ageo Central General Hospital, Ageo, Saitama 362-8588, Japan; y.kuramochi@kouaikai.jp (Y.K.); onoda@ach.or.jp (S.O.); nakajima.e@ach.or.jp (E.Y.); kimura.mas@ach.or.jp (M.K.); 4 Department of Physical Therapy, Health Science University, Fujikawaguchiko, Yamanashi 401-0380, Japan; kyosuke.fukuda@kenkoudai.ac.jp

**Keywords:** physical function, hospital stay, chronic kidney disease

## Abstract

Background: The relationship between hospitalization duration and physical function in patients with chronic kidney disease (CKD) has not been thoroughly investigated. This study aimed to determine whether assessment of physical function one week after hospitalization can predict the length of stay in patients with CKD. Methods: A retrospective study was conducted on hospitalized patients with CKD who underwent rehabilitation between March 2019 and March 2020. Physical function was evaluated using the Short Physical Performance Battery (SPPB), grip strength, and Barthel Index and analyzed alongside clinical data. Results: The mean age of the participants was 73.4 ± 11.9 years, with 92% having stage G4 or G5 CKD. Multivariate analysis revealed that the SPPB (β = −0.33, *p* < 0.01) at one week after admission was significantly associated with the length of hospital stay (R^2^ = 0.11, *p* < 0.02). Notably, in the subgroup of patients who were transferred to other facilities, the SPPB alone showed a strong association with the length of stay (β = −0.66, *p* < 0.03, R^2^ = 0.23, *p* < 0.05). Conclusions: The SPPB score in the early stages of hospitalization for patients with CKD was found to be a significant predictor of the length of stay, even after considering the eGFR and the Charlson Comorbidity Index. These findings may contribute to optimizing inpatient management and rehabilitation strategies for patients with CKD.

## 1. Introduction

Chronic kidney disease (CKD) is a global public health challenge with a worldwide prevalence that has been increasing in recent years. Many patients with CKD, particularly those with stages 4 and 5 (i.e., end-stage renal failure), are older and have a high risk of developing cardiac disease [1]. Therefore, many patients have cardiovascular complications on admission, which can lead to a significant decline in physical function during hospitalization.

Furthermore, patients with CKD are prone to muscle weakness and sarcopenia caused by decreased physical activity, accumulation of uremic substances, and acidosis [2,3,4]. These factors can lead to increased levels of uremic toxins, inflammatory cytokines such as TNF and IL-6, and other factors that contribute to muscle loss [2,5]. As a result, physical function is often further compromised during hospitalization.

Physical function is strongly associated with life expectancy. Studies have shown that walking speed and timed up-and-go tests predict 3-year mortality more accurately than renal function or commonly measured serum biomarkers [6]. Decreased physical function likely impairs the quality of life in this patient population, as it can lead to falls during hospitalization and decreased overall physical activity [6]. Therefore, the early and thorough evaluation of physical function may be crucial for improving quality of life and overall prognoses in hospitalized patients with CKD. However, improving physical function is highly time-consuming in older patients. Once a patient is hospitalized and bedridden, a significant hospital stay may be necessary before they can be discharged. The decline in physical function during hospitalization can contribute to a general decline in physical activity even after discharge. Physical inactivity and exacerbation of CKD are interrelated [7], and this decline in physical function during hospitalization creates a vicious cycle of hospitalization and discharge that needs to be addressed.

Personalized rehabilitation can help to prevent declines in physical function during hospitalization. Renal rehabilitation has recently been proposed and shown to be effective as a treatment for patients with CKD. We always initiate renal rehabilitation for hospitalized patients with CKD as soon as possible, within a few days. However, there are currently no clear indicators for selecting patients with declining physical function who would benefit from more intensive rehabilitation.

To the best of our knowledge, there are no studies that have discussed the physical function of hospitalized patients with CKD and not outpatients. This study aimed to evaluate physical function early during hospitalization for patients with CKD to determine whether it could predict the length of hospital stay and used the data to create a predictive model.

## 2. Materials and Methods

**Figure 1:**
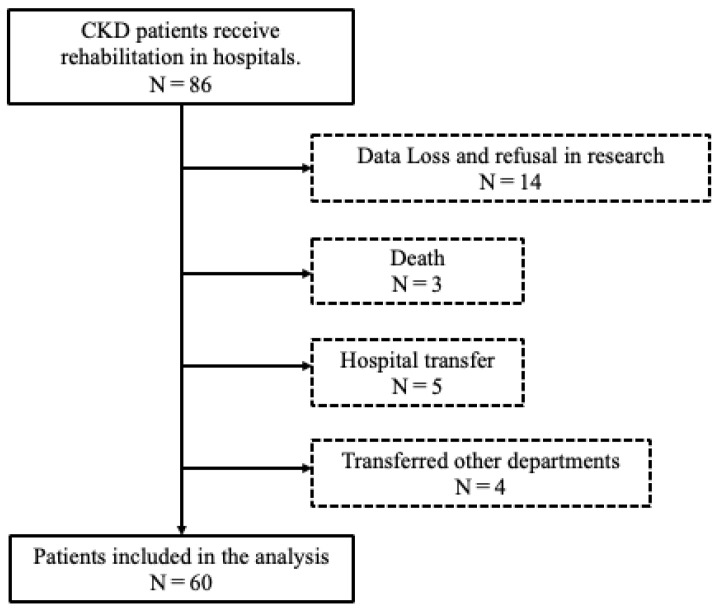
Patient selection for this study.

This retrospective study investigated the association between the Short Physical Performance Battery (SPPB) scores and hospitalization days. This study was approved by the Ethics Committee of Ageo Central General Hospital. The participants were 86 patients who were hospitalized for CKD overflow and underwent rehabilitation between March 2019 and March 2020. Patients who died during hospitalization, patients who could not be continuously evaluated by the end of rehabilitation, patients who were transferred to other departments due to complications, and patients who were transferred to other hospitals due to other acute diseases in hospitalization were excluded (Figure 1). This study was conducted at a large, well-equipped hospital that treats many hospitalized patients with CKD. At the institution, physical therapy for patients with CKD is a standard treatment shared by the CKD treatment teams. The CKD treatment team holds weekly conferences and collaborates with nurses, pharmacists, and the discharge support department. The nephrology department and rehabilitation department work closely together, with rehabilitation requests being made as soon as a patient is admitted. Physiotherapists typically intervene early after a patient has been admitted, performing gait checks during hospitalization.

The study measures included clinical data concerning age, sex, estimated glomerular filtration rate (eGFR), Charlson Comorbidity Index (CCI), CKD stage, and hospitalization length collected retrospectively from the hospital’s medical records. Physical function was measured using the SPPB, grip strength (GS), and the Barthel Index (BI), and it was assessed during daily living activities while hospitalized. The SPPB represents a comprehensive measure of lower limb function. The reliability and validity of the assessment have been proven in its clinical use [8,9,10].

### 2.1. Statistical Analysis

The basic statistics for the study were calculated for each of the items at 1, 2, and 3 weeks after admission. The means and standard deviations were extracted for age, sex, eGFR, CCI, CKD stage, SPPB, BI, and GS. Then, with the length of hospital stay as the dependent variable and the SPPB as the primary outcome, a multiple regression analysis was performed to examine the confounding effects of the eGFR and the CCI after adjusting for their values. Predictive accuracy was checked using the adjusted R^2^ value, and the VIF was used to assess multicollinearity. The strengths of relationships between the factors were determined using β values. A predictive equation for the length of hospital stay was then derived from the obtained predictors. JMP version 16 (SAS Institute Inc., Cary, NC, USA) was used for the analysis, with a statistical significance level of 5%. Graphs were generated using GraphPad Prism version 9.0 (GraphPad Software, Boston, MA, USA).

### 2.2. Ethics Approval and Consent to Participate

This study was conducted with the approval of the Ethics Committee of Ageo Central General Hospital (approval No. 866)

## 3. Results

Table 1 shows that the mean age of the total patient cohort at one week of hospitalization was 73.4 ± 11.9 years, with 28 males and 32 females. The patients who were hospitalized for 3 weeks tended to be older. The median eGFR value was low, and many of the patients had advanced CKD—with 92% being in CKD stages G4 or G5. A significant decline was observed in physical function at the SPPB values of 5.4 points and the GS of 14.4 kg. A significant decline in the ability to perform daily activities was observed at a BI value of 57.6 points.

Data concerning the patients discharged from the hospital by week are provided in Table 2. When the data were divided in this manner, no significant differences were observed for each parameter; however, the patients who remained hospitalized for up to three weeks tended to have lower SPPB scores. Table 3, Table 4 and Table 5 provide our multivariate analysis with hospital stay as the objective variable and the SPPB, eGFR, and CCI as the explanatory variables. In Table 3 Model 1 (R^2^ = 0.11, *p* < 0.02), the SPPB (β = −0.33, *p* < 0.01) after one week of physiotherapy intervention showed a significant association with the length of hospital stay. In Table 4 Model 2 (R^2^ = 0.01, *p* < 0.32), where only the patients discharged home were included, no significant differences were found for each indicator. By contrast, only the SPPB (β = −0.66, *p* < 0.03) showed significant differences in Table 5 Model 3 (R^2^ = 0.23, *p* < 0.05)—which only included the patients who were transferred to other hospitals. The predictive accuracy was higher for Model 3 than for Model 1 in terms of the adjusted R^2^.

**Figure 2:**
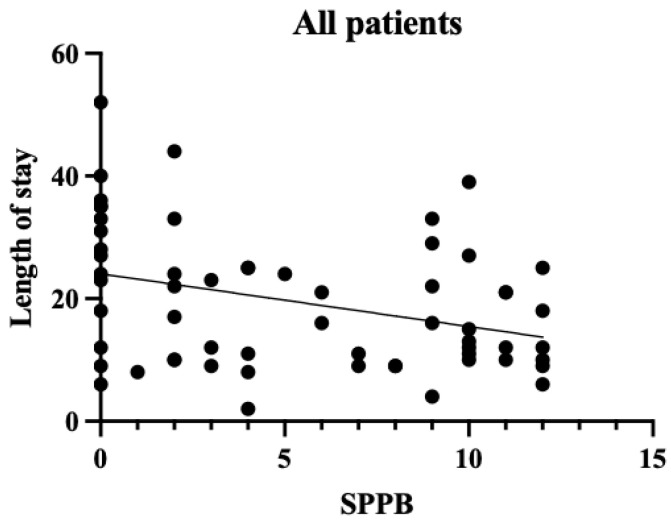
SPPB and length of stay in all patients. The vertical axis shows the number of days in the hospital and the horizontal axis shows the SPPB within a week. For all patients, there was a negative correlation between the SPPB and the length of hospital stay (*p* < 0.01).

**Figure 3:**
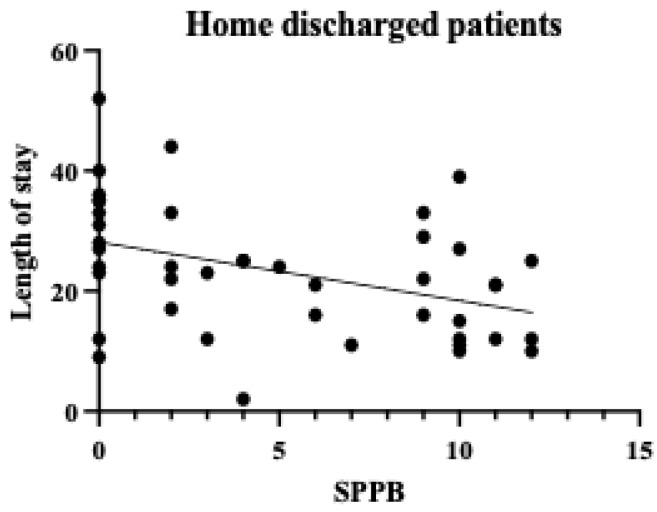
SPPB and length of stay in home-discharged patients. The vertical axis shows the number of days in the hospital and the horizontal axis shows the SPPB within a week. No significant differences were found.

**Figure 4:**
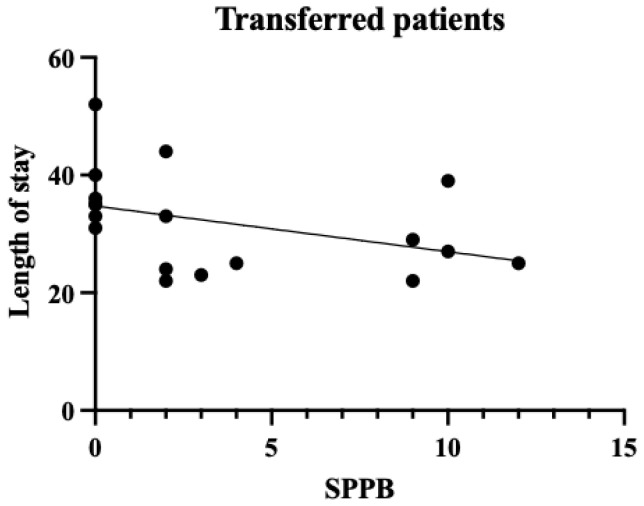
SPPB and length of stay in the transferred patients. The vertical axis shows the number of days in the hospital and the horizontal axis shows the SPPB within a week. For the transferred patients, there was a negative correlation between the SPPB and the length of hospital stay (*p* < 0.05).

A single regression correlation plot between the SPPB and the length of hospital stay is shown in Figure 2, Figure 3 and Figure 4. In the plot of patients who were transferred to other hospitals, those with low SPPB scores had a significantly longer length of stay than those who were discharged. Therefore, the SPPB was determined to be a significant predictor of the length of hospital stay, even when considering the eGFR and the CCI as confounding factors.

## 4. Discussion

CKD has become a major health challenge worldwide as the number of patients with the condition is increasing annually and has been reported to have reached 9.1–13.4% of the global population [11]. Patients with CKD often experience repeated hospitalizations. Once hospitalized [12], their physical function declines significantly as a result of the pathological characteristics of the disease, making it difficult for them to return home [13]. In addition, many patients with CKD have heart failure as a complication before hospitalization, which may lead to decreased physical function prior to admission [1]. Therefore, it is crucial to consider preventing physical function decline besides treating the conditions when planning the discharge of patients with CKD. From this perspective, the causal relationship between the length of hospital stay and the physical function was examined in patients with conservative CKD. The mean age of the patients in our cohort was relatively high, at 73.4 years, and the majority were in CKD stages G4–G5. In other words, the study population largely comprised older and severely ill patients. CKD stage and age have been reported to correlate with a decline in physical function [14]. Therefore, it should be considered that the study participants, being older and at more advanced CKD stages, may have had inherently lower levels of physical function. The SPPB has been shown to correlate strongly with social participation and the activities of daily living (ADL) [15] and is closely related to the ADL prognosis [16]. In this study, the mean SPPB score was 5.4 points, and the mean BI score was 57.6. These scores generally suggest a moderate decline in physical function corresponding to an ADL level requiring partial assistance in daily life. Therefore, many of the participants in this study were confirmed to have severe CKD and low baseline physical function. While older patients generally tend to experience declining physical function, it should be considered that this decline may be more pronounced when CKD is present, which is supported by this study’s findings. One week after admission, the physical function assessment results showed that the SPPB scores were significantly associated with the length of hospital stay. This suggests that early physical function assessment during hospitalization may represent an important indicator for predicting the subsequent length of stay. From the perspective of the treatment protocol for patients with CKD with volume overload, fluid removal is performed after hospitalization, and improvement in physical condition is expected within approximately 1 week. However, the results suggest that the recovery of physical function does not necessarily synchronize with improvements in pathological conditions. A strong correlation was observed between the SPPB and the length of hospital stay in the patients transferred to other hospitals (Table 5 Model 3), highlighting the importance of identifying which patients require more intensive rehabilitation as early as possible. Early appropriate interventions in pathological treatment have been reported to significantly affect patient outcomes [17]. Considering these results, it is crucial to conduct physical function assessments and rehabilitation interventions early, in parallel with pathological treatment, when managing inpatients with CKD. The use of objective physical function indicators such as the SPPB may allow for early judgment of the need for rehabilitation and the timely administration of appropriate interventions. Furthermore, the results of this study have important implications for discharge support and home medical care planning for patients with CKD. Figure 2 and Figure 3 show that many of our patients had the SPPB scores of 0, indicating that many hospitalized patients with CKD have low levels of physical function and ADL. This result is consistent with previous studies [13,18], which showed that elderly patients with CKD experience a significant decline in physical function. Figure 4 shows that among those who were discharged home, some patients with the SPPB scores of 0 points were discharged early, whereas those with such scores who were transferred to other facilities experienced longer hospital stays. These results suggest that support systems at home may significantly influence the length of hospital stay in patients with CKD. It is highly likely that these patients had already used a home care system before hospitalization. Patients with impaired physical function are likely to require more detailed discharge plans and continuous rehabilitation support. We believe that this will lead to an improvement in the amount of physical activity and quality of life. These results suggest that it is important to assess physical function early in hospitalization as a marker for predicting the length of stay for patients with stage G4–G5 end-stage CKD and that the SPPB may be a good indicator of this. Future challenges in this field include the development of early intervention programs based on the SPPB scores and verification of their efficacy. A limitation of this study is that the social background of the patients was not considered as a factor in their discharge. In terms of the number of cases, only the CCI and the eGFR were selected as the confounders that defined the length of hospital stay, but other confounders should also be considered. In particular, it should be noted that the excluded patients (those who were transferred to other hospitals due to acute illness or those who died) may have had more severe CKD and lower physical function. This potential bias in patient selection could have implications for our study results and their interpretation. Furthermore, it is important to note that this study can be considered a pilot study due to the limited available data and number of patients, which may not be sufficient to draw definitive conclusions. These limitations highlight the need for larger-scale studies to confirm and expand upon our findings. In future research, we would like to examine the factors related to the length of hospital stay by considering not only physical function, but also social aspects and various confounding factors, with a larger sample size, to enhance the robustness of our conclusions.

## 5. Conclusions

The results of this study suggest that conducting physical function assessments and rehabilitation interventions early, in parallel with the administration of treatments for the underlying pathology, may contribute to shortening hospital stays and improving the quality of life when managing inpatients with CKD. These findings have important implications for developing comprehensive treatment strategies for this vulnerable patient group.

## Figures and Tables

**Table 1:** Patient parameters.

		N = 60		
		Mean ± SD	Median	IR
Characteristics	Age	73.4 ± 11.9	76.0	67.0–82.0
	Sex (male/female)	28/32		
	Length of stay	19.4 ± 11.1	17.5	10.0–26.5
	eGFR	14.9 ± 18.6	8.6	5.4–14.7
	Charlson Comorbidity Index	7.8 ± 2.4	8.0	6.0–9.0
	CKD stage	G1A2 1 G2A3 2 G3bA3 2 G4A2 2 G4A3 10 G5A3 43	-	-
	RRT (implemented/not implemented)	29/31		
Physical function	SPPB	5.4 ± 4.5	4.0	0.3–10.0
	BI	57.6 ± 33.0	65.0	30.0–85.0
	Grip strength (kg)	14.4 ± 12.3	15.0	0–22.5

Note: eGFR: estimated glomerular filtration rate; CKD: chronic kidney disease; SPPB: Short Physical Performance Battery; RRT: renal replacement therapy; BI: Barthel Index; SD: standard deviation; IR: interquartile range.

**Table 2:** Patient parameters after admission.

		1 W (N = 4)		2 W (N = 22)		3 W (N = 34)		*p*-Value
		Mean ± SD	Median (IR)	Mean ± SD	Median (IR)	Mean ± SD	Median (IR)	
Variable	Age	80.4 ± 10.2	79 (70.7–90.2)	71.3 ± 13.6	76 (61.2–81.8)	74.1 ± 10.9	75.5 (68.5–87)	0.36
	Sex (male/female)	1/3		10/12		17/17		0.61
	Length of stay	4.5 ± 1.9	5 (2.5–6)	10.3 ± 1.5	10 (9–12)	27.1 ± 8.6	25 (21–33)	0.01 **
	eGFR	5.3 ± 1.6	5.1 (3.8–6.9)	20.5 ± 27.8	8.5 (4.6–21.2)	12.4 ± 9.4	9.2 (6–16.2)	0.16
	Charlson Comorbidity Index	6.8 ± 1	6.5 (6–7.8)	7.6 ± 2.6	7.5 (5–10)	8 ± 2.4	8(7–9.3)	0.55
	CKD Stage	G4A3 2 G5A3 2		G1A2 1 G2A3 2 G3bA3 2 G4A3 1 G5A3 16		G4A2 2 G4A3 7 G5A3 25		-
Physical function	SPPB	6.3 ± 5.3	6.5 (1–11.3)	6.7 ± 4.3	7.5 (2.8–10.3)	4.4 ± 4.4	2.5 (0–9)	0.12
	BI	51.3 ± 31.2	55 (20–78.8)	69 ± 26.3	80 (50–87.5)	51.3 ± 35.8	60 (10–85)	0.19
	Grip strength (kg)	15 ± 10.6	18.5 (3.8–22.3)	18.5 ± 14.9	18.5 (6.5–40.9)	11.9 ± 10.4	14 (0–20)	0.96

The 1 W, 2 W, and 3 W were defined as within 7 days, 8–14 days, and longer, respectively. ** < 0.01

**Table 3:** Model 1: all patients.

(N = 60)	β	*p*-Value	VIF
SPPB	−0.33	0.01 **	1.03
Charlson Comorbidity Index	0.15	0.22	1.05
eGFR	−0.1	0.4	1.03
Adjusted R^2^	0.11	0.02 *	-

* < 0.05; ** < 0.01.

**Table 4:** Model 2: home-discharged patients.

(N = 43)	β	*p*-Value	VIF
SPPB	−0.19	0.18	1.02
Charlson Comorbidity Index	0.1	0.49	1.07
eGFR	−0.16	0.3	1.08
Adjusted R^2^	0.01	0.32	-

**Table 5:** Model 3: transferred patients.

(N = 17)	β	*p*-Value	VIF
SPPB	−0.66	0.03 *	1.18
Charlson Comorbidity Index	0.08	0.76	1.22
eGFR	−0.08	0.75	1.26
Adjusted R^2^	0.23	0.05 *	-

* < 0.05. Factors affecting the length of hospital stay (days) by model. Note: eGFR: estimated glomerular filtration rate; SPPB: Short Physical Performance Battery; VIF: variance inflation factor.

## Data Availability

The datasets analyzed during this study are available from the corresponding author upon reasonable request.

## Abbreviations

The following abbreviations are used in this manuscript: chronic kidney disease (CKD), Short Physical Performance Battery (SPPB), estimated glomerular filtration rate (eGFR), Charlson Comorbidity Index (CCI), grip strength (GS), Barthel Index (BI).
